# Surgical treatment of obsessive compulsive disorders: Current status

**DOI:** 10.4103/0019-5545.55095

**Published:** 2009

**Authors:** Paresh K. Doshi

**Affiliations:** Stereotactic and Functional Neurosurgical Program, Jaslok Hospital and Research Centre, Mumbai, India

## INTRODUCTION

Surgery for intractable neuropsychiatric illness has generated considerable controversy for a variety of scientific, social, and philosophical reasons. Much of the controversy relates to the widespread and indiscriminate use of psychosurgery in the 1940s and 1950s when no effective psychotropic agents were available. With the introduction of chlorpromazine in 1954, effective medical management led to a rapid decline in surgery for mental illness. Despite the vast array of new, selective psychotropic medications available today, however, many neuropsychiatric illnesses remain refractory and, consequently, some patients remain severely disabled. These patients might be considered appropriate candidates for surgery if the overall result and level of functioning could be improved.

Obsessive-compulsive disorder (OCD) is a psychiatric disease characterized by anxiety-provoking thoughts (obsessions) leading to repeated, time-consuming behaviors (compulsions) that may or may not provide temporary relief. With an approximate prevalence of 2 to 3% of the general population and 0.6% in Indian population,[1] OCD is a debilitating disorder that can significantly affect nearly every aspect of a patient's life, and in some cases, lead to suicide.[2] In a meta-analysis of a database of the Food and Drug Administration, the annual suicide risk rate in OCD patients with minimal and no comorbidity, participating in a trial of selective serotonergic reuptake inhibitiors (SSRI) was 105/10000 and the annual suicide attempt risk was 1468/100000.[3] Although OCD has been recognized and studied in the psychiatric literature for nearly a century, only relatively recently has the disease been evaluated in a neuroscientific context. The application of functional imaging techniques, such as functional magnetic resonance imaging and positron emission tomography (PET) scans, to this patient population, coupled with advances in the safety and efficacy of functional neurosurgical intervention, has led to a renaissance of research in this area.

### Neurobiological model of obsessive-compulsive disorder

**Figure 1:**
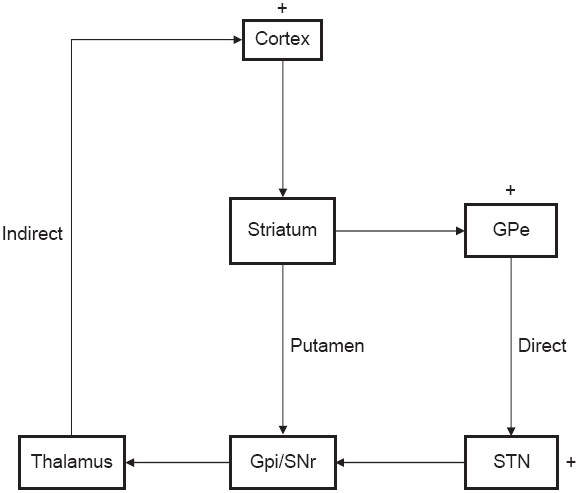
Diagram of CSTC circuit

There is a convergence of evidence implicating the corticostriatothalamocortical (CSTC) loop involving orbitofrontal (OFC) cortex, anterior cingulated cortex (ACC) and basal ganglia as central to the pathophysiology of OCD.[4–6] Two distinct routes are conceptualized from the striatum to the thalamus; the so-called “direct” and “indirect” pathways. The direct pathway projects from the cortex to the striatum to the internal segment of globus pallidus and substantia nigra to the thalamus and then back to the cortex. The indirect pathway is similar from the cortex to the striatum but then projects to the external segment of the globus pallidus to the subthalamic nucleus, before returning to the internal segment of the the globus pallidus/substantia nigra, there joining the direct pathway to the thalamus and projecting back to the cortex. Impulses transmitted via the direct pathway disinhibit the thalamus, presumably resulting in a release of behaviors as necessary for an adaptive function. Activity in the indirect pathway inhibits the thalamus, resulting in the cessation of ongoing behavioral routine. The prevailing theory on OCD suggests that a hitherto unknown primary striatal pathologic process underlies a relative imbalance favoring striatothalamic inhibition leading to hyperactivity within OFC and ACC, the caudate nucleus (CN) and the thalamus [Figure 1]. Besides this, prefrontal cortex, cingulated cortex, limbic circuit, OFC, hypothalamus and amygdala are the other structures that communicate with this primary circuits through various feedback loops. This forms the basis of various target sites for treating OCD. Guehl *et al.*, performed neuronal recordings from CN in three patients before Deep Brain stimulation surgery. These patients had very high Yale-Brown Obsessive Compulsive Scale (YBOCS). They found high discharge rate in the CN with variable interspike interval.[7]

### Imaging correlates of obsessive-compulsive disorder

Studies comparing volumes of specific brain regions in patients with OCD have often yielded conflicting and inconsistent results. Brain regions of interest have included the head of the CN, the OFC, and the ACC, as well as broader examinations of hemispheric and hippocampal asymmetry. With respect to the CN especially, studies have found increases,[8] decreases,[9,10] and no change[11] in volume between OCD patients and healthy controls on magnetic resonance image (MRI) scans. The source of this apparent discrepancy in the volumetric literature may be methodological in nature, with significant sample size variation, difficulty in acquiring adequate control subjects, and practical obstacles surrounding volume measurement all potentially impeding accurate and valid volume analysis. Furthermore, it has been suggested that the heterogeneity of OCD, a disease with several unique classifications and subtypes,[12] can be responsible for the inconsistent volume differences among various structures as reported in the literature. The other structure of interest is OFC, which is thought to sub serve motivational aspects of decision making and may have a role in behavioral adaptation to change, both of which have implications for OCD. Szesko *et al.*, found that the OFC volume was decreased in patients of OCD.[9]

### Functional imaging in obsessive-compulsive disorder

PET examines cerebral metabolism using flurodeoxyglucose. PET studies on patients with OCD confirm that elevated glucose metabolism occurs in the bilateral thalamus, caudate, and OFC regions. In PET scans of patients with OCD who exhibited compulsive hoarding as their primary symptom, lower rates of glucose metabolism were found in the anterior and posterior cingulate gyri when compared with patients who did not hoard and subjects without OCD.[6] The patients who did not hoard exhibited significantly greater activity in the bilateral thalamus and the CN. The study demonstrates differences between individuals with and without OCD, but it also indicates functional metabolic differences between patients with OCD, with patients who hoard showing different patterns of metabolism than patients who do not hoard. Similarly, in an another study of the patients who check, hypermetabolism was found in the putamen/globus pallidus, the thalamus, and the right inferior frontal cortex; in those who wash, the greatest activation was identified in the OFC, the cingulated gyrus (CG), and the ventrolateral prefrontal cortex. Such differences underscore the complexity of OCD and the inherent challenges that exist in using imaging to explore a disease that possesses several classifications and a diverse symptomatology.[11] A more recent study examining a broader range of studies used PET and SPECT[13] for measurement. The authors point to significant differences between patients with OCD and those without OCD, particularly in areas such as the orbital gyrus and the head of the CN. These findings also suggest that frontal and subcortical regions are possible key players in OCD; however, it is yet to be determined whether the hypermetabolic states detected are the causes or the consequences of the disorder. Although significant neuroanatomic and metabolic changes seem to exist, their identification must be viewed as starting points for additional investigation into the etiology of the disease as well as potential interventions. Notwithstanding these significant difficulties, the observations collected using functional imaging suggest that hypermetabolism of frontal regions occurs in OCD and consensus is growing regarding the metabolic states of the caudate and thalamic regions.[14]

### Surgical treatment for obsessive-compulsive disorder

Surgery for OCD is reserved for patients with the most severe cases of the disease, when pharmacological and psychotherapeutic alternatives have been exhausted. Although estimates in the literature vary, there is a relative consensus that a significant minority of OCD patients, from as low as 10% and up to 40%, are treatment refractory.[15–17] Some of these patients, who remain severely ill, are eligible for surgical intervention, given appropriate inclusion criteria and availability of required psychiatric and surgical expertise.

In 1891 the Swiss psychiatrist Gottlieb Burkhardt reported on surgical procedures with craniotomy in a series of six severely agitated patients, with success in three patients.[18] John Fulton from Yale observed the calming effect of frontal lobe surgery in chimpanzee, but at the same time cautioned about translating this animal experience to human trials. Egaz moniz, a neurologist from Lisbon along with Alemida Lima performed the first prefrontal leucotomy. He was later on awarded a Nobel Prize for his work in 1949. Walter Freeman a neuorpsychiatrist from US along with his surgical colleague James Watts performed frontal transection - later on came to be known as the famous “Ice Pick” surgery. It is another story that Watts later on severed ties with Freeman following his inadvertent use of this surgery. By 1954, more than 20 thousand surgeries were performed in US and similarly more than 10 thousand in UK. Ironically, it was a surgeon, Henri Laborit from Paris, who helped end the “golden age” of psychosurgery and usher in the pharamacologic age of psychiatry by noting the tremendous benefit Chlorproamzine offered in improving psychiatric disorders.

**Figure 2:**
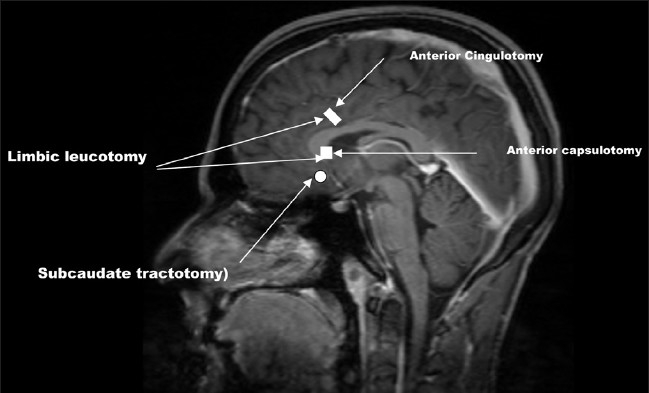
Targets of modern psychosurgery

The modern era of precision psychosurgery was brought in with invention of stereotactic equipment by Spiegel and Wycis.[19] The lesions became smaller and more precise, thus avoiding several side effects with larger lesions of prestereotactic era. During the last five decades, psychosurgery continued to be practiced at select centres. The four different targets currently being used are anterior capsule (AC), CG, Subcaudate tractotomy and Limbic leucotomy [Figure 2] Nucleus accumbens is another promising target for this surgery. AC and CG are the most popular and rewarding targets for OCD and hence will be discussed in detail. Two methods of surgery are employed for altering these targets. One involves performing lesion and the other involves stimulation of these targets using deep brain stimulation (DBS). In a lesion a radiofrequency unit is used to produce (destroy) a thermal lesion of calculated volume. This is permanent and irreversible. In DBS an electrode is implanted at the site of the target and current is delivered through a pacemaker to alter the signals emanating from the target. The pacemaker is implanted in the infraclavicular region and is connected by extension wires, tunneled subcuataneously to the electrodes that are implanted in the brain. The amount of current and thereby the stimulation/inhibition of the target site can be controlled by an external programmer. DBS offers an exceptional benefit of reversibility and titrability. DBS has been in use for now over four decades for pain and movement disorders. Recently US FDA approved the use of DBS for OCD.[20] Both this procedures are performed using stereotactic techniques which offer a high degree of accuracy (within 1-2mm).

### Cingulotomy

In 1937, the same year that Moniz[21] reported his initial experience with prefrontal lobotomy, Papez[22] postulated that a reverberating circuit in the human brain might be responsible for emotion, anxiety and memory. The components of this rudimentary limbic system included the hypothalamus, septal nuclei, hippocampi, mamillary bodies, anterior thalamic nuclei, CG and their interconnections. Laitinen showed that electric stimulation of the anterior cingulum and subcaudate region altered autonomic responses and anxiety levels in psychiatric patients.[23] PET studies have provided further evidence about the role of CG. In a small series of patients with chronic anxiety disorders and severe phobias, activation PET studies performed as the patients were presented with stimuli to recreate their fears demonstrated consistently increased regional cerebral blood flow in the ACC, OFC, left thalamus, and right CN.[24] Rauch *et al.*, reported atrophy of the caudate body in subjects who had undergone one or more cingulotomies approximately six months before the MRI studies.[25] Clinical observations suggest that OCD patients do not improve immediately after psychosurgery but that several weeks to months are required for positive clinical effects to manifest.[26]

Cingulotomy is carried out under mild sedation and local anaesthesia. MRI is used for defining target coordinates. Anterior CG is the preferred target site. Radiofrequency ablation is used to produce lesion. The resultant lesion involves 2.0-2.5 cm of entire thickness of anterior CG.

### Anterior capsulotomy/stimulation

**Figure 3:**
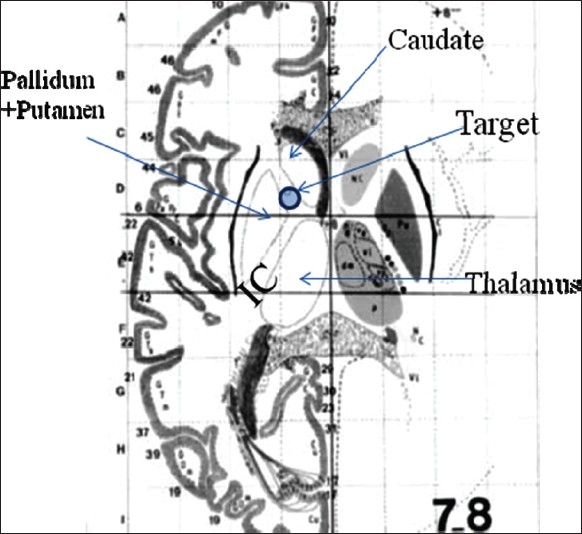
Capsulotomy/capsular stimulation target site

Capsulotomy was first described by Talairach in 1949 and popularized by Lars Leksell in Sweden.[27,28] The goal of anterior capsulotomy is to interrupt frontothalamic connections at the point where they converge in the anterior limb of the internal capsule, between the head of the caudate and putamen [Figure 3].

Anatomical studies confirm that the anterior limb of the internal capsule contains the anterior thalamic radiations (connecting the frontal lobes with the medial and anterior thalamic nuclei) and the prefrontal corticopontine tract.[29] Fiber connections between OFC and striatum also cross through internal capsule. Besides this internal capsule is surrounded by other important anatomical area like bed nucleus of stria terminalis, nucleus accumbens, ventral striatum which all form the part of the ventral striatopallidal complex. Interruption or modulation of these target sites forms the basis of OCD surgery. The advantage of DBS is that it can reversibly recruit neighbouring structures to improve the benefits of surgery without causing unwanted side effects. In 1999, Nutin *et al.*, demonstrated that electrical stimulation of the anterior limbs of the internal capsules induced beneficial effects in a patient with treatment-resistant OCD during the first minutes after the initiation of stimulation.[30] Although DBS has been applied at several locations along the rostral-caudal extent of the anterior limb of the internal capsule the target used most frequently includes the junction of the ventral anterior limb of the internal capsule and adjacent ventral striatum; known as the ‘VC/VS’.[31] DBS for OCD has undergone a parallel development in that the VC/VS target site has become more posterior as experience was gained, representing the greatest technical evolution from the beginning of this work in 1998. One hypothesis influencing this work is that more posterior locations may be more effective. Fibers within the cortico-striatal-thalamo-cortical (CSTC) networks hypothesized as central to the therapeutic effects of lesions or DBS[32] become more compact as they course posteriorly toward the thalamus, to which they connect via the inferior thalamic peduncle.[33,34] The VC/VS may thus represent a node of CSTC circuits that is readily targeted for modulation by DBS. The surgical procedure is similar to the cingulotomy. The target is the junction of the anterior capsule and the ventral striatum, within 1-2mm of the posterior border of the anterior commissure.

### Other target sites

Subcaudate tractotomy,[35] Limbic leucotomy and Gamma knife capsulotomy[36] are the other surgical options practiced by few groups. However, as their results are more or less similar and are less commonly practiced they will not be discussed. Of interest to note is that one group reported 89% success rate following limbic leucotomy with higher complication rates.[37] This may be due to more extensive lesions involved in limbic leucotomy (it is a combination of anterior cingulotomy and subcaudate tractotomy). Interested readers can refer to the references mentioned.

### Patient selection

The multicentre DBS trial was the most recent and systematic approach towards the surgical treatment of OCD. Four centres recruited 26 patients over eight year period. These criteria have been accepted by the “National Advisory Committee for Psychosurgery in India” which held its first meeting on 8^th^ March, 2009; for considering surgical interventions in OCD.

### Obsessive-compulsive disorder diagnosis and severity

Detailed patient screening, record review, interviews with treating clinicians and baseline assessments, including the Structured Clinical Interview for Diagnostic and Statistical Manual of Mental Disorders, 4^th^ edition,[38] should be used to assure that OCD is the primary diagnosis (the disorder judged by clinicians and patients as imposing the greatest burdens of symptom and functional impairment). OCD has to be of at least 5 year's duration. YBOCS symptom intensity in the ‘severe’ range was required (score of 28 or more). OCD should be judged to cause marked functional impairment with a Global assessment of Functioning (GAF) score of 45 or less.

### Treatment resistance

This is defined as adequate trial (≥3 months) with maximally tolerated doses of at least three serotonin reuptake inhibitors (SRIs), one of which has to be clomipramine. Trials combining an SRI with additional medications (including a neuroleptic and a benzodiazepine) should also be tried. All patients were required to have had behavior therapy, defined as a minimum of 20 sessions of therapist-guided exposure and response prevention. Patients who attempt behavioral therapy but who demonstrate marked intolerance to it (in the therapist's judgment) are eligible.

### Exclusion criteria

Patients are excluded if there is a history of a current or past psychotic disorder, a manic episode within the preceding 3 years, any current clinically significant neurological disorder or medical illness (except for tic disorders) or any clinically significant abnormality on preoperative MRI, any labeled DBS contraindication and/or inability to undergo pre-surgical MRI, history of substance abuse or dependence or a clinical history of severe personality disorder.

### Independent review

At each center desirous of performing OCD surgery a committee including psychiatrists not connected with the surgery, neurologist and neurosurgeon should review the clinical histories, baseline evaluations and the consent process.

### Other rating scores

Hamilton Rating Scale for Depression (HAM-D), Beck Depression inventory (BDI) and Hamilton Rating Scale for Anxiety (HAM-A) should also be performed.

## RESULTS

In a retrospective report of 198 patients undergoing cingulotomy for various psychiatric disorders, Ballantine noted 56% improvement in patients with OCD. There is generally a delay of 3-6 months in the onset of beneficial effect after cingulotomy. Using subjective rating scales, approximately 60% to 70% patients show significant improvement. When more objective clinically validated rating scales are used to assess outcome, approximately 30-45% patients are considered to be responders.[39–41] The average duration of follow-up was two years. The results are better with larger or repeat lesions. Minor symptoms of headache, low-grade fever, and nausea are common after cingulotomy but generally last less than 24-48 hours. Transient unsteady gait, dizziness, confusion, urinary retention, and isolated seizure can occur; although mild and self limiting, these symptoms may last up to several weeks. Permanent significant behavioral or cognitive decline has not been reported after cingulotomy.[42] In a review of stereotactic cingulotomy, Cosgrove and Rauch[25] described OCD treatment experience of one major centre. In 800 cingulotomies performed over a 40-year period at Massachusetts General Hospital, there were no deaths and only two infections reported. Change *et al.*, performed pre and post operative SPECT imaging in patients undergoing cingulotomy.[43] They noted preoperative hyperperfusion in the anterior CG and post operative hypo perfusion in anterior CG and OFC following improvement in the symptoms.

Leksell reported that 50% of OCD patients responded to capsulotomy. In another study, Bingley found that 25 out of 35 patients (78%) were either symptom-free or much improved an average of 35 months after thermocapsulotomy.[44] Mindus and Jenike retrospectively reviewed all cases of capsulotomy reported by the early 1990s. They judged that 64% of 213 patients for whom adequate information was available could be considered responders.[45] Anecdotally, after surgery, adherence to and success of behavioral therapy seemed much enhanced in responders.

In the multicentre DBS for OCD trial the mean preoperative YBOCS was 34 ± 0.5. The postoperative improvement was gradual, similar to that observed in lesional surgeries. At three months there were 50% responders who improved to 61.5% at the last follow up and if one considers the last group of patients (who were implanted electrodes at the new target site, i.e. the posterior part of anterior limb of IC), the improvement was seen in more than 75% patients. The GAF increased from an average of 34 to 59. At last follow-up, work, school or homemaking functioning was described as fair or good in 21 of the 25 patients. Capacity for independent living was considered fair or good in 20 of 25 patients. There was also significant improvement in the co-morbid anxiety and depression. The improvement in depression and anxiety is observed earlier than the improvement in OCD symptoms. As discussed earlier DBS involves titration of optimal parameters for symptom control. During this period there is an immediate improvement in mood and anxiety providing a clue to selecting the right contact point and stimulation parameters.

DBS is associated with a slightly higher incidence of complications that include hemorrhage, infection and hardware failures. In the above multicentre study there were two incidence of hemorrhage (not significant) and one incidence of hardware complications. Patients reported worsening of symptoms when their stimulators were switched off, this improved upon restarting the stimulators.

O15-PET imaging in a subset of patients from this series found that acute high frequency DBS increased perfusion in OFC, ACC, striatum, pallidum and thalamus compared to control conditions.[46] Fluorodeoxyglucose positron emission tomography (FDG-PET) imaging in a different subset of these patients found that metabolism in the subgenual CG, measured before surgery, was directly correlated with the extent of OCD improvement during DBS.[47]

## CONCLUSION

In 1997 the Journal of Clinical Psychiatry published a supplement in the Expert Consensus Guidelines Series entitled ‘Treatment of obsessive-compulsive disorder’.[48] The guideline states ‘In the adult with extremely severe and unremitting OCD, neurosurgical treatment to interrupt specific brain circuits that are malfunctioning can be very helpful’. However, it seems that surgical therapy for intractacble OCD may be underused. It is 12 years since this guidelines and today we are at a very fortunate period where our understanding of OCD has improved and so has the surgical therapy. The modern neurosurgical practice, including DBS, has made surgical interventions less risky. The reversibility and titrability which DBS provides would help the fence sitters also to take a surgical decision. Surgery, however, is not the end of the disease story. It would need preoperative and postoperative active participation from the psychiatrists to manage these patients, as the responsibility and commitment towards the patient care would increase following surgery.
